# Monocular complex amplitude imaging via a polarization-multiplexed liquid-crystal-lens-informed Fourier neural network

**DOI:** 10.1093/nsr/nwaf561

**Published:** 2025-12-09

**Authors:** Liu Li, Minghao Liao, Yixin Zhang, Zishuai Zeng, Shuai Wang, Wenhe Jia, Jing Zhang, Bohan Zhang, Yiying Dong, Dapeng Zhang, Fei Zhang, Yuanmu Yang

**Affiliations:** State Key Laboratory of Precision Measurement Technology and Instruments, Department of Precision Instrument, Tsinghua University, Beijing 100084, China; Henan Key Laboratory of Diamond Optoelectronic Materials and Devices, Key Laboratory of Integrated Circuits, Ministry of Education, School of Physics, Zhengzhou University, Zhengzhou 450052, China; State Key Laboratory of Optical Field Manipulation Science and Technology, Institute of Optics and Electronics, Chinese Academy of Sciences, Chengdu 610209, China; College of Materials Sciences and Opto-Electronic Technology, University of Chinese Academy of Sciences, Beijing 100049, China; State Key Laboratory of Precision Measurement Technology and Instruments, Department of Precision Instrument, Tsinghua University, Beijing 100084, China; State Key Laboratory of Precision Measurement Technology and Instruments, Department of Precision Instrument, Tsinghua University, Beijing 100084, China; State Key Laboratory of Precision Measurement Technology and Instruments, Department of Precision Instrument, Tsinghua University, Beijing 100084, China; State Key Laboratory of Precision Measurement Technology and Instruments, Department of Precision Instrument, Tsinghua University, Beijing 100084, China; State Key Laboratory of Precision Measurement Technology and Instruments, Department of Precision Instrument, Tsinghua University, Beijing 100084, China; State Key Laboratory of Precision Measurement Technology and Instruments, Department of Precision Instrument, Tsinghua University, Beijing 100084, China; State Key Laboratory of Precision Measurement Technology and Instruments, Department of Precision Instrument, Tsinghua University, Beijing 100084, China; State Key Laboratory of Optical Field Manipulation Science and Technology, Institute of Optics and Electronics, Chinese Academy of Sciences, Chengdu 610209, China; College of Materials Sciences and Opto-Electronic Technology, University of Chinese Academy of Sciences, Beijing 100049, China; State Key Laboratory of Optical Field Manipulation Science and Technology, Institute of Optics and Electronics, Chinese Academy of Sciences, Chengdu 610209, China; College of Materials Sciences and Opto-Electronic Technology, University of Chinese Academy of Sciences, Beijing 100049, China; State Key Laboratory of Precision Measurement Technology and Instruments, Department of Precision Instrument, Tsinghua University, Beijing 100084, China

**Keywords:** complex amplitude imaging, liquid-crystal lens, physics-informed neural network

## Abstract

The success of data-driven deep learning in computational imaging is often constrained by the need for extensive labeled datasets. Recent progress in physics-informed neural networks has mitigated this issue by integrating analytical physical models, allowing data-free training. However, for challenging imaging tasks, such as to simultaneously acquire complex amplitude light-field information, the weak physical constraints of conventional imaging hardware largely limit the spatiotemporal imaging resolution. Here, we propose an extremely simple yet powerful monocular camera for complex amplitude imaging based on a liquid-crystal (LC)-lens-informed Fourier neural network. Combining a polarization-multiplexed bifocal LC lens with a polarization image sensor, the camera acts as a polarization phase-shifting radial shearing interferometer. Without any labeled data, the LC-lens-informed Fourier neural network can reconstruct the complex amplitude of a variety of scenes from captured polarization images in a single shot with high fidelity. We experimentally demonstrate the reconstruction of wavefront aberrations involving 136 Zernike modes with a phase accuracy of *λ*/35 as well as static hologram retrieval and dynamic monitoring of air flow and flame fields. This complementary hardware-algorithm framework offers a promising pathway for developing compact, versatile and high-performance complex amplitude imaging systems for adaptive optics, hologram reconstruction and material diagnosis applications.

## Teaser

A monocular camera using a liquid-crystal-lens-informed Fourier neural network enables single-shot, high-fidelity complex amplitude imaging without labeled data.

## INTRODUCTION

Artificial intelligence has driven groundbreaking advancements in computational imaging [1–3], with data-driven neural networks emerging as key solutions for inverse problems. Their universal mapping capabilities, combined with proper hardware, have enabled the precise characterization of multidimensional light fields, such as polarization [4], spectral [5] and phase [6]. However, the training of neural networks typically relies on large-scale, high-precision labeled datasets to ensure reconstruction fidelity [7], significantly increasing data-acquisition costs while limiting generalizability across diverse imaging scenarios. To eliminate the need for labeled data, the physics-informed neural network (PINN) [8,9] has emerged as a promising paradigm for solving ill-posed problems. PINNs employ a physical model to map the network’s predictions to theoretical measurements, thereby creating a physics-based loss function that guides the neural network to learn the forward mapping process through backpropagation. As coherent optical fields can be physically modeled by using diffraction equations, the PINN has demonstrated exceptional capability in tackling phase retrieval [10], with successful applications in optical metrology [11,12], aberration correction [13] and quantitative phase imaging [14,15].

The challenging task of complex optical field reconstruction, requiring the simultaneous recovery of amplitude and phase, necessitates rigorous physical models from optical systems to enable reliable full-field characterization for practical applications. However, estimating complex optical fields from a single-shot-intensity image is highly ill-posed. As a result, simple physical models such as free-space diffraction [11] or scattering propagation [16] can only reconstruct pure-phase objects, failing to drive the neural network to decouple and recover both the phase and the intensity. To enhance the physical constraints for complex amplitude imaging, multiple-diversity-intensity measurements are typically required, achieved by varying the imaging parameters, including the image-plane location [17,18], illumination angle [19] or illumination wavelength [14]. Increasing the measurement frames compresses the solution space, enabling more accurate network updates and superior reconstruction. Nonetheless, such diversity in measurement inevitably compromises either temporal resolution or form factor due to the finite space-bandwidth product of conventional imaging systems.

Recent advancements in flat optics [20–23] have provided a compelling solution to these challenges. A flat optical component with subwavelength features enables the unprecedented manipulation of vectorial light fields [24–26] and supports versatile multiplexing strategies [27–30]. This paradigm enhances information throughput while preserving system simplicity, enabling diversity measurements through single-shot acquisition. Recent studies have demonstrated qualitative phase visualization leveraging nonlocal metasurfaces [31–34]. To achieve quantitative measurement, the transport-of-intensity equation (TIE) has been implemented by capturing multiple defocused images using metasurfaces [35–38], although this approach is limited to weakly absorbing phase objects. Breaking new ground, spatial-polarization multiplexing with more sophisticated meta-devices has enabled quantitative phase gradient imaging [39–41] and even complex amplitude imaging [42,43] through lateral shearing interferometry. However, as functionalities become increasingly diversified, metasurfaces present challenges in production costs, broadband capability, aperture size and diffraction efficiency. More importantly, such excessive multiplexing also compromises the field of view (FOV) and spatial resolution, significantly degrading reconstruction fidelity. A high-fidelity computational imaging framework capable of fully harnessing the multifunctional capabilities of flat optical components still remains an open challenge.

Here, we propose and experimentally demonstrate a general computational complex amplitude imaging framework by using a liquid-crystal (LC)-lens-informed Fourier neural network. The extremely compact monocular imaging system, composed of a bifocal LC lens and a polarization image sensor, encodes the target complex amplitude into Stokes parameters via polarization phase-shifting radial shearing interferometry, avoiding the FOV reduction associated with spatial multiplexing. Without any labeled data, this physical imaging model drives the Fourier neural network to reconstruct the complex amplitude directly from single-shot polarized images without spatial-resolution loss. Compared with metasurfaces, the high-energy-efficiency LC lens enables low-cost mass production while avoiding FOV reduction with excessive spatial multiplexing. Experimental results demonstrate that the system exhibits strong generalization capabilities, enabling high-precision Zernike aberration diagnostics, hologram reconstruction and dynamic complex amplitude imaging of air flow and flame combustion.

## RESULTS

### Complex amplitude imaging model

**Figure 1.**
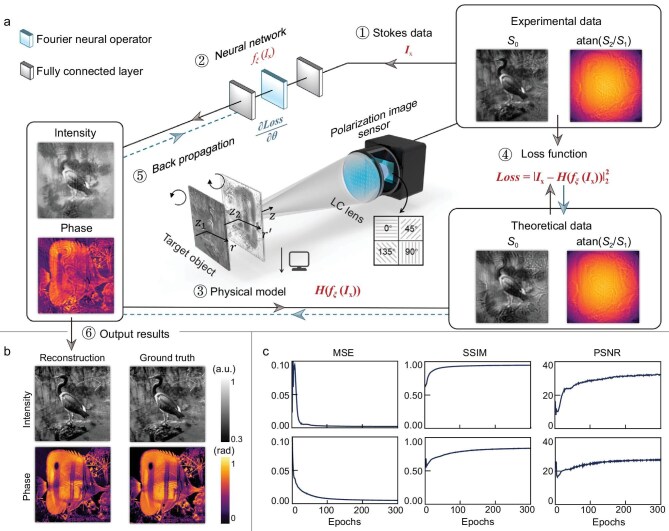
Complex amplitude imaging framework with the LC-lens-informed Fourier neural network. (a) Flowchart of complex amplitude reconstruction based on the LC-lens-informed Fourier neural network and LC lens. The insets include the complex amplitude output by the neural network and the theoretical Stokes data, both from the 25th training epoch. (b) Simulated intensity and phase reconstruction results, along with their corresponding ground truth. (c) Evolution of image-evaluation metrics for reconstructed intensity and phase with iteration number. MSE, mean squared error; SSIM, structural similarity index measure; PSNR, peak signal-to-noise ratio.

The complex amplitude imaging framework of the LC-lens-informed Fourier neural network is schematically illustrated in Fig. 1a. An optimized bifocal LC lens, designed with distinct focal lengths *f*_L_ and *f*_R_ for left-circularly polarized (LCP) and right-circularly polarized (RCP) light, captures LCP and RCP images at different imaging magnifications (*M*_L_ and *M*_R_) along the optical-axis *z*-direction within planes *r*-*z*_1_ and *r’*-*z*_2_. By leveraging the polarization phase-shifting radial shearing interferometry [44], the system creates a well-constrained physical model that independently encodes the target intensity and phase into separate Stokes parameters.

Specifically, the target complex amplitude *A*e*^iφ^* is located at the object plane *r*-*z*_1_ where the captured LCP light field *U*_LCP_ = *A*e*^iφ^* is in focus. Due to the difference in focal lengths, the imaging system captures the RCP light field *U*_RCP_ as the defocused complex amplitude at plane *r*-*z*_2_. The diffraction of *A*e*^iφ^* from *z*_1_ to *z*_2_ can be modeled by using the angular spectrum method as:


(1) \begin{eqnarray*}
{U}_{{\mathrm{RCP}}} &=& A^{\prime}{{\mathrm{e}}}^{i\varphi ^{\prime}}\\
&=& {\mathcal{F}}^{ - 1}\left[ {\mathcal{F}\left[ {A{{\mathrm{e}}}^{i\varphi }} \right]\exp \left[ { - i\pi \lambda ({z}_1 - {z}_2)k_r^2} \right]} \right]{\mathrm{,}}\\
\end{eqnarray*}

where *λ* is the incident wavelength, $\mathcal{F}$ denotes the 2D Fourier transform and ${\mathcal{F}}^{ - 1}$ its inverse, and *k*_r_ represents the spatial frequency in the radial direction. To avoid the random polarization phase difference between LCP and RCP light, the illumination beam is set to be linearly polarized and the direction of polarization can be arbitrary.

Combined with a polarization image sensor, the interference patterns of LCP and RCP light are projected onto four polarization detection channels at 0°, 45°, 90° and 135°, respectively. The complex amplitude information is contained in the computed Stokes parameters *S*_0_ = *I*_0°_ + *I*_90°_, *S*_1_ = *I*_0°_ – *I*_90°_ and *S*_2_ = *I*_45°_ – *I*_135°_. Such a forward-imaging physical model *H* [·], mapping the target complex amplitude to the captured Stokes parameters, can be expressed as (see Note S1):


(2) \begin{eqnarray*}
H{\mathrm{\ [}}A{{\mathrm{e}}}^{i\varphi }] &=& \left[ {\begin{array}{@{}*{1}{c}@{}} {{\mathrm{atan}}\left( {\frac{{{S}_{\mathrm{2}}}}{{{S}_{\mathrm{1}}}}} \right)}\\ {{S}_{\mathrm{0}}} \end{array}} \right]\\
&=& \left[ {\begin{array}{@{}*{1}{c}@{}} {\varphi \left( {{M}_{\mathrm{L}}r} \right) - \varphi ^{\prime}\left( {{M}_{\mathrm{R}}r^{\prime}} \right)}\\ {{{\left| {A{\mathrm{(}}{M}_{\mathrm{L}}r{\mathrm{)}}} \right|}}^{\mathrm{2}}{\mathrm{\ + \ }}{{\left| {A^{\prime}{\mathrm{(}}{M}_{\mathrm{R}}r^{\prime}{\mathrm{)}}} \right|}}^{\mathrm{2}}} \end{array}} \right].
\end{eqnarray*}

Thus, the constructed physical model incorporates free-space diffraction at a defocus distance |*z*_1_–*z*_2_| and polarization phase-shifting radial shearing interferometry with a shearing ratio of *M*_L_/*M*_R_, providing strong physical constraints for the complex amplitude reconstruction.

To reconstruct the target complex amplitude light field, the constructed physical model is employed to drive the training process of the neural network. The neural network takes the captured experimental data *I*_x_ as input and outputs the reconstructed complex amplitude *f*(*I*_x_), where *f*_ξ_(·) represents the mapping process of the neural network. Subsequently, the reconstructed complex amplitude is transferred into the forward physical model to compute the theoretical data *H*(*f*_ξ_(*I*_x_)), which, together with the experimental data, are used to construct the loss function. Thereafter, the neural network parameters ξ are updated via backpropagation, completing one training epoch. This process is repeated until the loss function converges, finally yielding the reconstructed complex amplitude light-field distribution, as illustrated in Fig. 1b.

Our framework demonstrates distinct advantages over conventional Gerchberg–Saxton (GS) [45] and TIE [35] algorithms. By leveraging high-dimensional Stokes parameters rather than scalar intensity stacks, it provides a more constrained information basis for phase retrieval. Furthermore, the neural network’s capacity to learn sophisticated inverse mappings allows it to be compatible with intricate physical models, surpassing the limitations of the fixed physical models used in traditional techniques, such as the Fresnel diffraction model in GS and the paraxial Helmholtz equation in TIE. Ultimately, the seamless integration of the optical LC-lens encoder with the computational decoder enables single-shot, high-fidelity complex amplitude imaging—a capability fundamentally beyond the reach of multi-frame methods.

To improve the reconstruction speed, the neural network architecture has been optimized to incorporate a Fourier neural operator [46] (FNO) and fully connected layers. Unlike the convolutional neural network (CNN) [47], the FNO offers enhanced global information-extraction capabilities and lower computational complexity, facilitating the learning of mappings between infinite-dimensional function spaces (see Note S2). While the FNO has traditionally been used for solving partial differential equations, we find them exceptionally efficient for computational complex amplitude imaging. This efficiency likely stems from the physical consistency between the Fourier transform within the FNO and the diffraction propagation of coherent light fields.

Ultimately, benefitting from the improved neural network architecture and enhanced physical model, the framework achieves the rapid simultaneous reconstruction of both phase and intensity. The number of Fourier modes in the FNO was set at 200, satisfying the Nyquist–Shannon sampling theorem [48] while maintaining reconstruction efficiency (see Note S3). For a sampling of 400 × 400 pixels, the complex amplitude is reconstructed in just 15 seconds through 300 epochs by using a computer equipped with a NVIDIA GTX 1650 Graphics Processing Unit (GPU), as illustrated in Fig. 1c. In contrast, the method based on CNN and free-space diffraction [11] can only reconstruct the phase distribution and requires 10 minutes through 10 000 epochs with a resolution of 256 × 256 pixels and a more powerful NVIDIA Quadro P6000 GPU. The reconstruction efficiency, defined as the ratio of inference time to image width, has improved by two orders of magnitude compared with CNN-based methods (see Note S4). To comprehensively evaluate the reconstruction performance, three metrics—mean squared error (MSE), peak signal-to-noise ratio (PSNR) and structure similarity index measure (SSIM)—were employed to quantify the quality of the reconstructed images. All image-evaluation metrics rapidly converged with increasing training epochs.

To decouple the contributions of the FNO and the physical model, a U-Net was also tested under the same conditions (see Note S5). The U-Net-based reconstruction required 3000 epochs and ∼171.7 seconds to converge to a level equivalent to the FNO network, which is an order of magnitude slower in reconstruction efficiency. Therefore, the significant gain in reconstruction efficiency is attributed to the joint utilization of our physical model and the FNO architecture.

### LC-lens design and characterization

**Figure 2.**
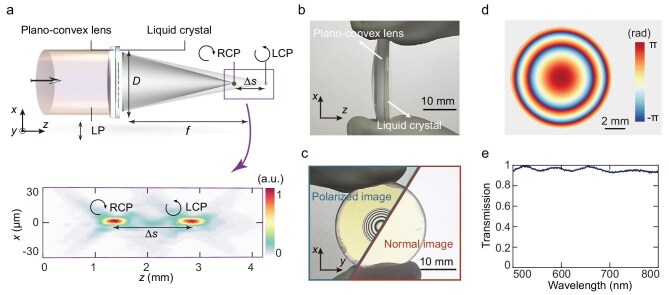
LC-lens design and characterization. (a) Schematic of the LC lens that focuses the circularly polarized light with different focal lengths, where *f* is the focal length of the plano-convex lens, *D* is the diameter of the LC lens and ∆*s* is the separation distance. The inset below shows measured PSFs along the *z*-direction. LP, linear polarized. (b) Side view of the LC lens. (c) Optical images of the LC lens under polarized and unpolarized illumination. (d) Geometric phase distribution of the LC layer. (e) Wavelength-dependent transmission of the LC layer.

The schematic of a static LC lens with distinct focal lengths for orthogonal circularly polarized light is shown in Fig. 2a. With a linearly polarized plane wave incident, the focal points of LCP and RCP light are axially separated by ∆*s*. To generate such a point spread function (PSF) at *λ* = 633 nm, we designed a LC lens composed of a single layer of LC molecules tightly bonded to a plano-convex lens. The geometric phase induced by the rotation of the LC molecules results in opposite phase distributions for LCP and RCP light. By further incorporating the converging propagation phase of the refractive lens, the focal points of LCP and RCP light are separated along the optical axis near the original focal point of the plano-convex lens. To achieve axial shifts of ±∆*s*/2 for LCP and RCP light, the designed geometric phase *ϕ*_LC_ (*x, y*) of the LC molecules is derived as (see Note S6):


(3) \begin{eqnarray*}
{\phi }_{{\mathrm{LC}}}\!\left( {x,y} \right) = \frac{\pi }{\lambda }\left( {\frac{f}{{\sqrt {{x}^2 + {y}^2 + {f}^2} }} - 1} \right)\Delta s{\mathrm{,}}
\end{eqnarray*}

where *f* = 75 mm is the focal length of the plano-convex lens, Δ*s* = 1.4 mm is the separation distance of the LCP and RCP focal points, corresponding to focal lengths of *f*_L_ = 75.7 mm and *f*_R_ = 74.3 mm for LCP and RCP light, respectively. With the target object placed at a finite distance of 2*f*_L_, the separation distance Δ*s* determines the radial shearing ratio *M*_L/_*M*_R_ and the defocus diffraction distance |*z*_2–_*z*_1_| in the physical model, with *M*_L/_*M*_R_ ≈ 1 + 2Δ*s*/*f*_L_ and |*z*_2–_*z*_1_| ≈ 4Δ*s*, respectively (see Note S6). As a system hyperparameter, Δ*s* has been optimized through parameter scanning to ensure compatibility with the neural network. For the LC lens shown in Fig. 2, with a diameter *D* = 10 mm, the numerical aperture is 0.067. Under collimated light illumination, the allowed maximum FOV of the single-lens imaging system is identical to the aperture size of the LC lens. Increasing the aperture size improves both spatial resolution and FOV.

For the proof of concept, as shown in Fig. 2b, a LC layer with a 10-mm-diameter patterned area was fabricated on a 1-inch fused silica substrate by using the photoalignment technique (see Note S7). Subsequently, it was tightly bonded to a plano-convex lens. Polarized and unpolarized images of the fabricated LC lens are shown in Fig. 2c. The geometric phase distribution of the LC layer, optimized by using the ray-tracing method (Zemax OpticStudio) to further match the geometry of the plano-convex lens, is illustrated in Fig. 2d (see Note S8). The fabricated LC layer, with a pixel size of 5.4 μm, can provide a maximum phase gradient of 0.39 rad/μm, significantly exceeding the required phase gradient of 0.0061 rad/μm. The measured separation distance in the PSFs align with the designed separation distance (inset of Fig. 2a). Also, the measured diffraction limit for a single LCP/RCP focus (5.83 μm) closely matches the theoretical diffraction limit of the plano-convex lens (5.79 μm). As shown in Fig. 2e, the LC layer exhibits high transmission across a broad wavelength range and the measured diffraction efficiency of the assembled LC lens is ≤94.23% at a wavelength of 633 nm (see ‘Methods’ and Note S9). Compared with the metalens made of silicon or titanium dioxide nanopillars, LC-based devices support large-aperture fabrication at a relatively low cost, which is advantageous for achieving spatial-multiplexing-free complex amplitude imaging with relatively large FOV and high spatial resolution (see Note S10 for detailed comparisons).

### LC-lens-based complex amplitude imaging

**Figure 3.**
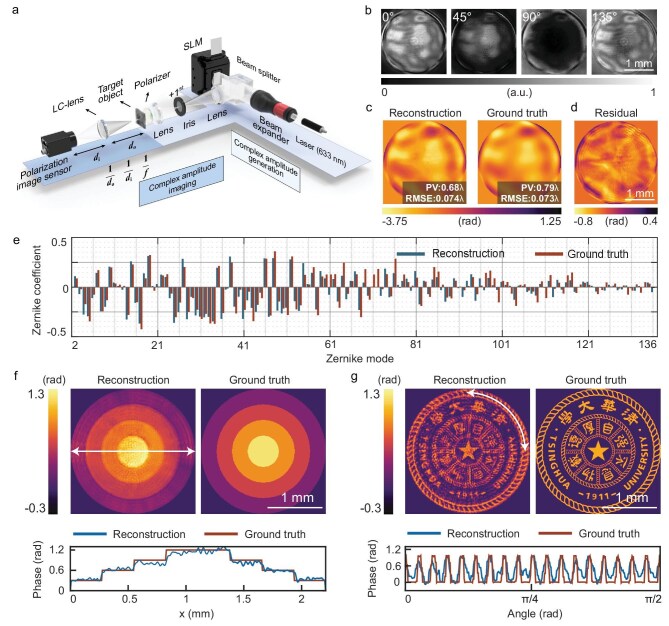
Pure-phase detection using the LC-lens-informed Fourier neural network. (a) Schematic of the complex amplitude generation and imaging system. (b) Captured interference patterns with the polarization channel along 0°, 45°, 90° and 135°, respectively. (c) Reconstructed phase distribution and its corresponding ground truth. (d) Residual distribution between measured result and ground truth. (e) Histogram of decomposed Zernike coefficients versus Zernike mode. (f) Reconstructed step-like phase and (g) Tsinghua University emblem phase distributions, together with their corresponding ground truth. The lower panel illustrates the phase distribution along the white solid line in the reconstruction results.

To demonstrate the versatility of the complex amplitude imaging system across multiple scenarios, we use the phase-only spatial light modulator (SLM) to generate various complex amplitude light-field distributions based on computer-generated holography (CGH) [49], as shown in Fig. 3a (for more details, see ‘Methods’). The fabricated LC lens was then integrated with a polarization image sensor for complex amplitude imaging. To mitigate potential mismatches between the forward physical model and experimental parameters, a plane wave was employed to calibrate the complex amplitude imaging system (see Note S11). Compared with conventional interference-based systems [50], this compact, reference-free system is expected to exhibit lower sensitivity to environmental disturbances such as mechanical shocks, air turbulence and temperature fluctuations.

As a prototype, the object and image distances of the complex amplitude imaging system were set to *d*_o_ = *d*_i_ = 2*f*_L_, enabling clear imaging of LCP light with an imaging magnification of *M*_L_ = 1. The corresponding radial shearing ratio and defocus diffraction distance were *M*_L/_*M*_R_ = 1.04 and |*z*_2–_*z*_1_| = 5.60 mm, respectively. To experimentally validate the performance of the proposed complex amplitude imaging system, we first measured the Zernike aberration polynomials [51], which are essential for quantifying and correcting optical aberrations in wavefront analysis. A wavefront aberration distribution composed of Zernike polynomials from the 2nd to the 136th orders was loaded onto the SLM, with Zernike coefficients following a normal distribution across the mode numbers to simulate the aberration characteristics of real optical systems. The interferometric images in the four polarization channels were directly captured by using the polarization camera, as shown in Fig. 3b. Subsequently, the 2D wavefront aberration reconstructed using the LC-lens-informed Fourier neural network, together with the corresponding ground truth from the computer-generated bitmap loaded onto the SLM, is illustrated in Fig. 3c. In this configuration, our system functions similarly to a Shack–Hartmann wavefront sensor [52] and provides precise measurements of wavefront aberrations at specific image planes, making it particularly suitable for optical component inspection [53] and adaptive optics applications [54].

To benchmark the system performance, we utilized two widely adopted metrics in adaptive optics—the peak-to-valley (PV) value and the root mean square error (RMSE)—to characterize the wavefront aberrations. The PV value, defined as PV = max(*φ*) – min(*φ*), was measured to be 0.68*λ*, reflecting the extreme cases of wavefront distortion. The RMSE for the entire measurement area, defined as ${\mathrm{RMSE = }}\sqrt {\frac{{\mathrm{1}}}{N}\mathop \sum \nolimits_{n{\mathrm{ = 1}}}^N {{| {\varphi ( n ){\mathrm{ - }}{\varphi }_{{\mathrm{avg}}}} |}}^{\mathrm{2}}} $, was 0.074*λ*, where *N* is the number of spatial sampling points and *φ*_avg_ is the average phase value. The measured PV and RMSE values characterize the extreme and global fluctuations of the wavefront aberration, which align well with the ground truth. The phase accuracy, defined as the RMSE of the residual (Fig. 3d), is 0.029*λ* (∼*λ*/35). To provide a more comprehensive physical analysis of their composition, the wavefront aberrations were decomposed into Zernike coefficients across different modes, as shown in Fig. 3e. The reconstructed Zernike aberration coefficients from the 2nd to the 136th orders show a high degree of consistency with the ground truth.

Moreover, the proposed system is capable of measuring both slowly varying wavefront aberrations and abrupt phase distributions—a feature beyond the reach of traditional Shack–Hartmann wavefront sensors [52]. As shown in Fig. 3f and g, we demonstrate the reconstruction of a staircase phase target and the Tsinghua University logo, achieving phase accuracies of 0.014*λ* and 0.059*λ*, respectively, where the phase accuracy is defined as the RMSE of the residuals between the measured results and the ground truth. The insets of Fig. 3f and g show that the reconstructed radial and tangential step-phase distributions both align closely with the ground truth.

**Figure 4.**
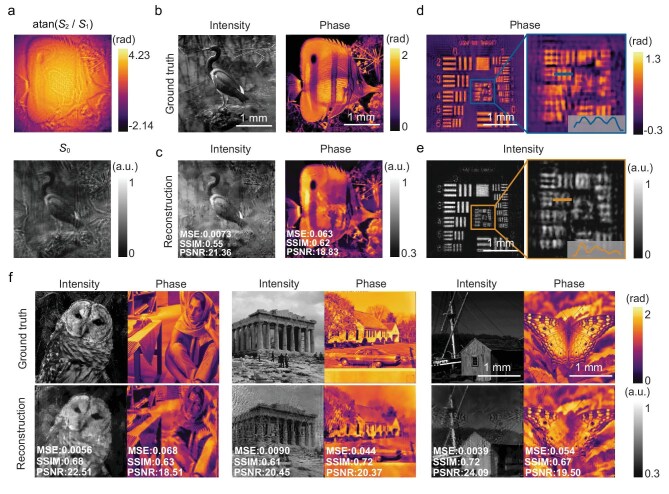
Complex amplitude imaging using the LC-lens-informed Fourier neural network. (a) Captured Stokes parameters images with the polarization image sensor. (b) Reconstructed complex amplitude and (c) its corresponding ground truth. (d, e) Reconstructed 1951 USAF (d) phase and (e) intensity resolution target. (f) Reconstructed diverse complex amplitudes and their corresponding ground truths.

To further testify the generalization capability of the monocular complex amplitude imaging system, we prepared a wide variety of independent and highly complex phase and intensity distributions as the inputs. The Stokes parameters were calculated from the polarized intensity captured by the polarization image sensor, as shown in Fig. 4a. The reconstructed complex amplitude (Fig. 4b) was denoised by using the nonlocal means filter [55] (Fig. 4c). The filtering algorithm was only an isolated post-processing step applied to the final output of the neural network and therefore does not affect the framework’s generalization capability. The relatively low SSIM of the reconstructed image is partly due to the challenges in generating high-quality complex amplitude fields based on CGH, which include the nonuniform response of the SLM [56], the aperture limitation of the 4-*f* filtering system and the inherent speckle noise in coherent fields [57]. Based on the same optical configuration (see Note S12), we directly captured a clear intensity image of the generated optical field as a reference ground truth, with metrics of MSE = 0.0065, SSIM = 0.66 and PSNR = 21.87. In comparison, the evaluation metrics of the reconstructed intensity image (MSE = 0.0073, SSIM = 0.55, PSNR = 21.36) are very close to the reference ground truth.

Subsequently, the US Air Force (USAF)-1951 phase (1.2-rad height) and intensity resolution targets were loaded to evaluate the spatial resolution of the monocular complex amplitude imaging system. To match the minimum linewidth of 15.88 μm (corresponding to Group 2, Element 3) achievable by the SLM, the aperture of the LC lens was restricted to 3.7 mm. As shown in Fig. 4d and e, the reconstructed intensity and phase distributions clearly resolve up to Group 2, Element 3, achieving a minimum spatial resolution of 31.76 μm, consistently with the ideal imaging conditions (see Note S13). Thus, despite the use of a non-ideal bifocal imaging PSF, the neural network still enables complex amplitude reconstruction without resolution degradation.

The complex amplitude reconstructions of a wide range of random scenes are shown in Fig. 4f, demonstrating that the neural network can reconstruct various complex amplitude fields without hyperparameter tuning, which is unattainable when using traditional data-driven neural networks.

**Figure 5.**
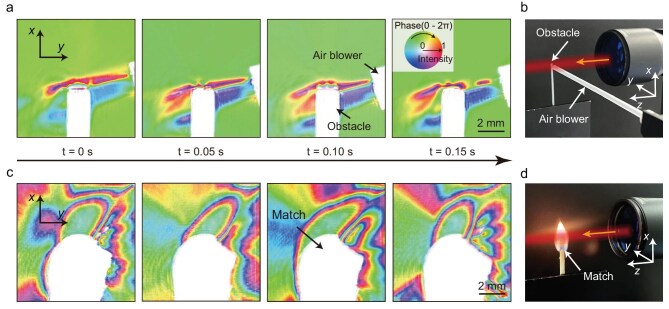
Dynamic complex amplitude imaging for air flow and flame combustion. (a, c) Selected frames of the measured complex amplitude distribution of the (a) air flow and (c) flame combustion processes captured at a video frame rate of 20 Hz. (b, d) The experimental setup in which the laser beam passes through the (b) air flow and (d) flame fields.

As the complex amplitude image can be obtained in a single shot, the system is particularly suitable for monitoring the dynamic evolution process. Experimentally, we employed a 4-*f* imaging system cascaded with the LC lens to expand the FOV of dynamic complex amplitude imaging (see Note S14). Here, we measured the complex amplitude images of air flow passing through a fixed obstacle, recorded at a frame rate of 20 Hz, as shown in Fig. 5a and b and Movie S1. In addition, we captured the complex amplitude variations within the flame field during the match combustion at a frame rate of 20 Hz, as shown in Movie S2 and Fig. 5c and d. The frame rate for dynamic capture is limited only by the polarization image sensor, whereas the complex amplitude reconstruction for each frame is performed offline, requiring ∼15 seconds per frame on our current system.

## CONCLUSION

In summary, by integrating a LC lens, a commercial polarization camera and a labeled-data-free PINN, we have developed an extremely simple monocular camera for single-shot complex amplitude imaging, applicable to diverse scenarios, while breaking the trade-offs between physical volume and spatiotemporal imaging resolution. The system achieves high-fidelity complex amplitude imaging with *λ*/35 phase accuracy and no spatial-resolution loss, while the reconstruction speed is improved by two orders of magnitude compared with CNN-based methods. More importantly, current flat optical components often operate as black boxes within data-driven neural networks, preventing the full utilization of their modulation potential. Therefore, this interpretable framework for the deep integration of flat optical components and neural networks is essential for advancing real-world intelligent multidimensional light-field sensing systems, free from the constraints of labeled datasets. Beyond this, the LC layer can be specifically designed and integrated with various imaging lens systems, including, but not limited to, microscopes and endoscopes, to generate different focal lengths for LCP and RCP light, thereby enabling a broader range of applications. We also envision that tailored design of the neural network architecture, integrated with complex amplitude capture, could achieve an aberration-corrected camera [58] and single-shot far-field synthetic aperture super-resolution imaging [59].

## METHODS

### LC-lens fabrication

The LC lens consists of a plano-convex lens (CX10612-633, Lbtek Optics) and an LC layer patterned on a 1-inch fused silica substrate. The fabrication process involves several steps, as detailed in Note S7. Initially, the substrate underwent cleaning with acetone followed by ultraviolet (UV) ozone treatment to enhance the surface wettability. An SD1 solution was spin-coated to create the photoalignment layer, which was then baked for stabilization. Using a digital-micromirror-device-based microlithography system, the photoalignment layer was exposed for alignment recording. Subsequently, the LC solution was spin-coated and UV-cured under 365-nm unpolarized light. This coating and curing process was repeated four times to achieve the desired half-wave thickness, ensuring the optimal efficiency of the LC lens.

### PSF and diffraction efficiency measurement

To measure the PSF of the fabricated LC lens, we constructed a setup as shown in Note S9. A linearly polarized laser beam (HSXYD1250, Huashang Laser) at 633 nm was incident on the LC lens. The focal spots of the LC lens were subsequently magnified through a 20× microscope system (MPLNAPO20XVIR, Mitutoyo). The PSF was acquired by moving the LC lens along the optical axis.

To estimate the diffraction efficiency of the LC lens, a circularly polarized laser beam at 633 nm was expanded and then truncated by a 10-mm aperture to match the diameter of the LC lens. Subsequently, the incident laser was focused onto a power meter (PM122D, Thorlabs) by using a plano-convex lens (CX10612-633, Lbtek Optics) with a 200-μm pinhole placed in front of the power meter to precisely capture the power of the focal spot. By spatially scanning the position of the power meter, the maximum power near the focal point was determined and recorded as the incident power *P*_inc_. Finally, the above procedure was repeated to measure the optical power *P*_LC_ focused by the LC lens. The diffraction efficiency of the metalens was estimated as *η* = *P*_LC_/*P*_inc_.

### Complex amplitude generation and imaging setup

For the complex amplitude generation system, a 633-nm diode laser beam was expanded and illuminated on the SLM (X15213-01, Hamamatsu). The designed CGH pattern with a blazed grating phase was loaded onto the SLM with a resolution of 722 × 722 pixels and a pixel size of 12.5 μm. Subsequently, a 4-*f* system (with lens focal lengths of 100 and 30 mm) filtered out the +1-diffraction order to avoid the zero-order diffraction spot. Finally, the conjugate image plane of the 4-*f* system generated the target complex amplitude field with a spatial size of 2.76 × 2.76 mm^2^.

For the complex amplitude imaging system, the complex amplitude light field was captured by using a commercial polarization image sensor (MV-CH050-10UP, Hikvision) through the LC lens. The polarization camera is equipped with a polarization-sensitive complementary metal oxide semiconductor sensor (IMX250LQR, SONY). Each sub-pixel of the sensor has a size of 3.45 μm, with 2 × 2 sub-pixels forming a macro-pixel for polarization detection along 0°, 45°, 90° and 135°, respectively. At a 1:1 imaging magnification, the LC-lens-based imaging system achieved a resolution of 400 × 400 pixels for the complex amplitude field generated by the SLM.

For the dynamic complex amplitude imaging system, the experimental setup consists of the fabricated LC lens cascaded with a 4-*f* system to enable dynamic complex amplitude imaging with an expanded FOV of 9.2 × 9.2 mm^2^, as shown in Note S14.

### Configuration of the neural network

The LC-lens-informed Fourier neural network consists of three FNO layers and two fully connected layers, as shown in Note S2, which is implemented by using Python version 3.8, Pytorch framework version 2.2.2 and CUDA version 12.6. The Adam optimizer was employed with a learning rate of 0.06. The model was trained on a computer equipped with an AMD Ryzen 3700X Central Processing Unit (CPU) and an NVIDIA GTX 1650 GPU.

## Supplementary Material

nwaf561_Supplemental_Files

## Data Availability

The Python codes used in this paper are available at https://github.com/THUMetaOptics/LC-lens. All relevant data are available in the main text, in the Supporting Information or from the authors.
