# Supplementary material for: Monocular complex amplitude imaging via a polarization-multiplexed liquid-crystal-lens-informed Fourier neural network
Source: Natl Sci Rev. 2025 Dec 9;13(2):nwaf561. doi: 10.1093/nsr/nwaf561 (PMC12839537; doi:10.1093/nsr/nwaf561)
Supplement: nwaf561_Supplemental_Files [file nwaf561_supplemental_files.zip › LiuLi_LCLensQPI__SupplementaryInformation_NSR_check.pdf]

Supplementary Information:

Monocular complex amplitude imaging via a polarization-multiplexed liquid-crystal-lens-informed Fourier neural network

Liu Li[1,4,#], Minghao Liao[2,3,#], Yixin Zhang[1,#], Zishuai Zeng[1], Shuai Wang[1], Wenhe Jia[1], Jing Zhang[1], Bohan Zhang[1], Yiying Dong[1], Dapeng Zhang[2,3], Fei Zhang[2,3,*], Yuanmu Yang[1,*]

[1]State Key Laboratory of Precision Measurement Technology and Instruments, Department of Precision Instrument, Tsinghua University, Beijing 100084, China
[2]State Key Laboratory of Optical Field Manipulation Science and Technology, Institute of Optics and Electronics, Chinese Academy of Sciences, Chengdu 610209, China
[3]College of Materials Sciences and Opto-Electronic Technology, University of Chinese Academy of Sciences, Beijing 100049, China
[4]Henan Key Laboratory of Diamond Optoelectronic Materials and Devices, Key Laboratory of Integrated Circuits, Ministry of Education, School of Physics, Zhengzhou University, Zhengzhou 450052, China

[#]These authors contributed equally.
*zf@ioe.ac.cn
*ymyang@tsinghua.edu.cn

Note S1: Forward physical model of the complex amplitude imaging system.

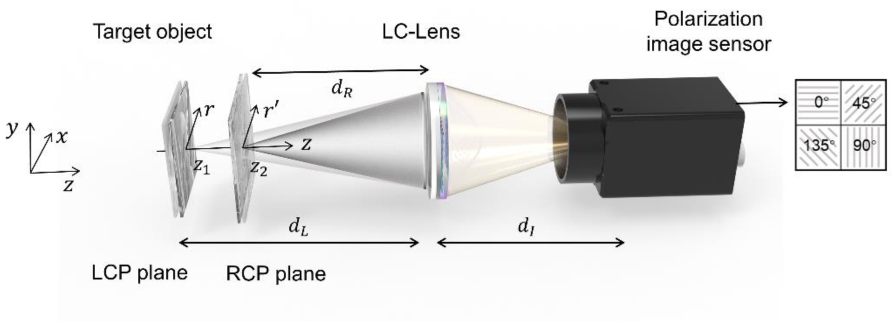

Figure S1 | Schematic diagram of the radial shearing interferometry based on polarization phase shift.

The target object with complex amplitude U_{obj} is characterized by a two-dimensional amplitude distribution $A(x, y)$ and phase distribution $\varphi(x, y)$, which, under linearly polarized illumination, can be decomposed into left-circularly polarized (LCP) and right-circularly polarized (RCP) light fields. As illustrated in Fig. S1, the complex amplitude imaging system based on an LC-lens, exhibits distinct focal lengths f_{LCP} and f_{RCP} for LCP and RCP light, allowing clear imaging of the LCP and RCP light fields at object distances d_{LCP} and d_{RCP}. The imaging relationship can be expressed as,

$$\frac{1}{d_{\text{LCP}}} + \frac{1}{d_I} = \frac{1}{f_{\text{LCP}}}, \tag{S1}$$

$$\frac{1}{d_{\text{RCP}}} + \frac{1}{d_I} = \frac{1}{f_{\text{RCP}}}, \tag{S2}$$

where d_{I} is the image distance, the LCP and RCP fields at object distances d_{LCP} and d_{RCP} exhibit different imaging magnifications, given by $M_{\text{LCP}} = d_{\text{I}} / d_{\text{LCP}}$ and $M_{\text{RCP}} = d_{\text{I}} / d_{\text{RCP}}$, respectively.

The target object is located at the LCP object plane (r, z_1), and the LCP light field U_{LCP} in the laboratory cylindrical coordinate system (r, z) can be expressed as,

$$U_{\text{LCP}} = U_{\text{obj}} = \frac{\sqrt{2}}{2}\begin{bmatrix}1\\ i\end{bmatrix} A(r)\exp[i\varphi(r)]. \tag{S3}$$

The imaging system captures the RCP light field U_{RCP} as the defocused complex amplitude at plane r-z_2. The diffraction of $Ae^{i\varphi}$ from z_1 to z_2 can be modeled by the angular spectrum method as,

$$U_{\text{RCP}} = A'e^{i\varphi'} = F^{-1}\left[F\left[Ae^{i\varphi}\right]\exp\left[-i\pi\lambda(z_1 - z_2)k_r^2\right]\right], \tag{S4}$$

where λ is the incident wavelength, $\mathcal{F}$ denotes the 2D Fourier transform and $\mathcal{F}^{-1}$ its inverse, and k_{r} is the spatial frequencies in the radial direction.

The interference pattern is then captured by the polarization image sensor. By applying the Jones matrix of the four linear micro-polarizers (0°, 45°, 90°, and 135°) at each macro-pixel of the polarization image sensor, the interference light intensity can be calculated as,

$$I_{0°} = \left|\begin{bmatrix}1 & 0\\ 0 & 0\end{bmatrix}(U_{\text{LCP}} + U_{\text{RCP}})\right|^2 = I_{\text{LCP}} + I_{\text{RCP}} + 2\sqrt{I_{\text{LCP}} I_{\text{RCP}}}\cos(\Delta\varphi), \tag{S5}$$

$$I_{45^\circ}=\left|\frac{1}{2}\begin{bmatrix}\sqrt{2} & \sqrt{2}\\ 0 & 0\end{bmatrix}(U_{\mathrm{LCP}}+U_{\mathrm{RCP}})\right|^2 = I_{\mathrm{LCP}}+I_{\mathrm{RCP}}+2\sqrt{I_{\mathrm{LCP}}I_{\mathrm{RCP}}}\cos\left(\Delta\varphi-\frac{\pi}{2}\right), \quad \text{(S6)}$$

$$I_{90^\circ}=\left|\begin{bmatrix}0 & 0\\ 0 & 1\end{bmatrix}(U_{\mathrm{LCP}}+U_{\mathrm{RCP}})\right|^2 = I_{\mathrm{LCP}}+I_{\mathrm{RCP}}+2\sqrt{I_{\mathrm{LCP}}I_{\mathrm{RCP}}}\cos(\Delta\varphi+\pi), \quad \text{(S7)}$$

$$I_{135^\circ}=\left|\frac{1}{2}\begin{bmatrix}\sqrt{2} & -\sqrt{2}\\ 0 & 0\end{bmatrix}(U_{\mathrm{LCP}}+U_{\mathrm{RCP}})\right|^2 = I_{\mathrm{LCP}}+I_{\mathrm{RCP}}+2\sqrt{I_{\mathrm{LCP}}I_{\mathrm{RCP}}}\cos\left(\Delta\varphi+\frac{\pi}{2}\right), \quad \text{(S8)}$$

where $I = |U_{\mathrm{LCP/RCP}}|^2$ is the light intensity and $\Delta\varphi = \varphi - \varphi'$ is the phase difference $\Delta\varphi$ between LCP and RCP light. Combining Eqs. (S5)-(S8), the phase difference $\Delta\varphi$ can be calculated as,

$$\Delta\varphi = \varphi(M_{\mathrm{LCP}}r) - \varphi'(M_{\mathrm{RCP}}r') = \operatorname{atan}\left(\frac{S_2}{S_1}\right) = \operatorname{atan}\left(\frac{I_{45^\circ} - I_{135^\circ}}{I_{0^\circ} - I_{90^\circ}}\right), \quad \text{(S9)}$$

where $S_1 = I_{0^\circ} - I_{90^\circ}$ and $S_2 = I_{45^\circ} - I_{135^\circ}$ are the Stokes parameters.

The intensity component of the complex amplitude is contained within Stokes parameter S_0, which can be derived as,

$$S_0 = I_{0^\circ} + I_{90^\circ} = |A(M_{\mathrm{LCP}}r)|^2 + |A'(M_{\mathrm{RCP}}r')|^2. \quad \text{(S10)}$$

Thus, Eqs. (S9) and (S10) form the forward imaging physical model $H[\cdot]$, which maps the target complex amplitude to the captured Stokes parameters, and $H[\cdot]$ can be expressed as,

$$H[Ae^{i\varphi}]=\begin{bmatrix}\operatorname{atan}\left(\frac{S_2}{S_1}\right)\\ S_0\end{bmatrix}=\begin{bmatrix}\varphi(M_{\mathrm{LCP}}r) - \varphi'(M_{\mathrm{RCP}}r')\\ |A(M_{\mathrm{LCP}}r)|^2 + |A'(M_{\mathrm{RCP}}r')|^2\end{bmatrix}. \quad \text{(S11)}$$

Note S2: Architecture of the Fourier neural network

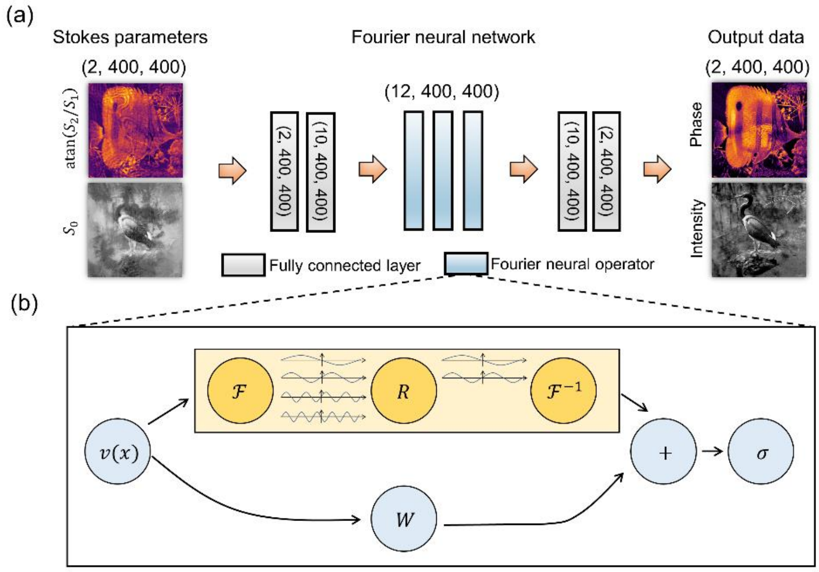

Figure S2 | Architecture of the Fourier neural network. **a**, The full architecture of the Fourier neural network. **b**, The full architecture of the Fourier neural operator.

The specific architecture of the Fourier neural network is illustrated in Fig. S2(a). The network takes two-channel Stokes parameter as input data, which is first processed by a fully connected layer to expand the channel dimension to match the subsequent Fourier operator layers. The network then incorporates three Fourier neural operator (FNO) layers, each configured with 200 Fourier modes and 12 hidden channels, to efficiently extract global features. Finally, two additional fully connected layers map the output to two-channel reconstructed intensity and phase distributions.

As depicted in Fig. S2(b), the FNO layer begins by applying the Fourier transform to the input variable $v(x)$. A linear transformation layer R is then applied to the lower modes and filters out the higher modes. The output variable is subsequently obtained through the inverse Fourier transform, and a bias term W from a local linear transformation is added, yielding the final output variable σ of the FNO layer.

The Fourier neural network exhibits significant advantages over the U-net convolutional neural network primarily in three aspects. First, U-net neural network relies on convolutional operators that focus on local information, making it sensitive to noise. In contrast, FNO is a global operator that efficiently extracts the overall features of the input data. By filtering out higher modes through Fourier transformation, it achieves enhanced robustness. Second, FNO demonstrates superior physical consistency in phase retrieval. The entire imaging process, from the reconstructed complex amplitude to the captured Stokes parameters, can be described by the Fresnel diffraction equation based on Fourier transforms. Also, the Fourier transform in FNO employs trigonometric functions as basic functions, which naturally align with continuous equations derived from Fourier transforms, providing a consistent expression of the diffraction process. Finally, in computational efficiency, FNO shows

a significant advantage in time complexity. For a single Fourier filtering layer, the time complexity is $O(N\log(N))$, where N represents the number of Fourier modes, typically half the width of the input image. In contrast, the time complexity of a single convolutional layer in U-net is $O(M^2 \cdot K^2)$, where M is the width of the input image and K is the size of the convolutional kernel, resulting in higher computational complexity than FNO. Additionally, the symmetric architecture of U-net requires more network layers, further increasing the overall computational complexity.

Note S3: Impact of the number of Fourier Modes on reconstruction performance

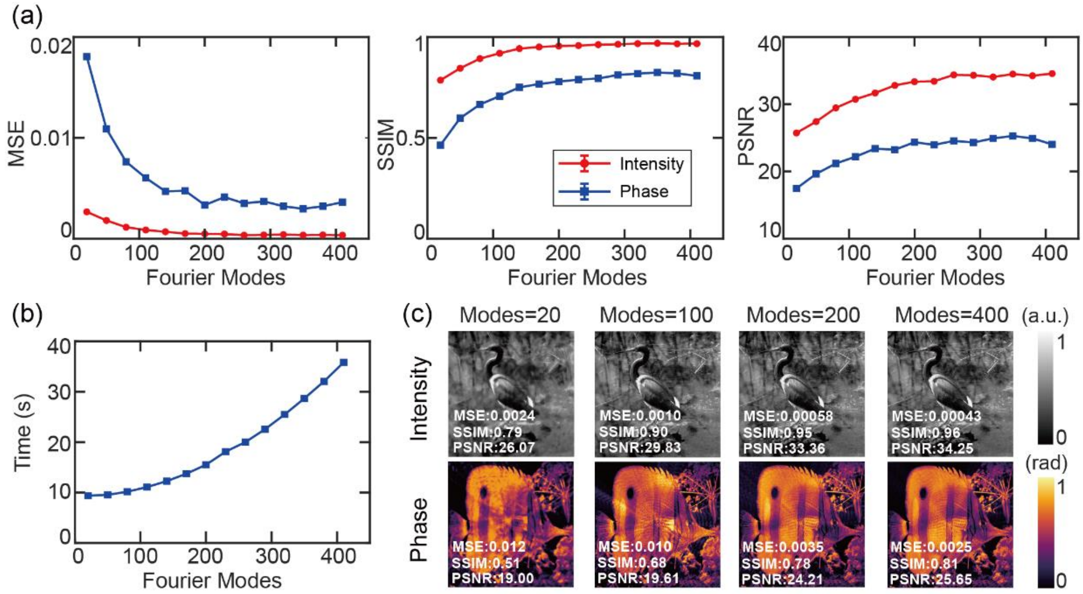

Figure S3 | Reconstructed complex amplitude with different numbers of Fourier modes. (a) Image evaluation metrics of the reconstructed complex amplitude as a function of the number of Fourier modes. (b) Reconstruction time as a function of the number of Fourier modes. (c) Reconstructed complex amplitude images for different numbers of Fourier modes.

As a key hyperparameter, the Fourier modes presents a trade-off: while more modes enhance reconstruction quality, they also increase computational time. In the FNO neural network, the number of Fourier modes corresponds to the maximum spatial frequencies retained after filtering. According to the Nyquist-Shannon sampling theorem[1], the retained Fourier modes must exceed half of the image sampling points to accurately reconstruct the image without losing high-frequency information. Therefore, we set the Fourier modes to 200 for the 400 × 400 pixels target images.

We also performed a parameter sweep to evaluate the impact of this hyperparameter. As shown in Fig. S3a, the complex amplitude reconstruction quality shows no significant improvement beyond 200 Fourier modes. Meanwhile, the reconstruction time increases with the number of modes (Fig. S3b). The reconstruction results with different Fourier modes are provided in Fig. S3c for qualitative comparison. Consequently, 200 Fourier modes is the optimal choice for achieving the highest reconstruction efficiency and reconstruction quality.

Note S4: Comparison between the convolutional neural network and the Fourier neural network

Method	Neural network	Target object	Image size	Epochs	Time (s)	Reconstruction efficiency (s/pixel)
**Free space diffraction[2]**	CNN	Phase	256 × 256	10000	600	2.34
**Scattering medium propagation[3]**	CNN	Phase	64 × 64	120	270	4.21
**Digital holography[4]**	CNN	Phase	512 × 512	7000	600	1.17
**Fourier ptychographic microscopy[5]**	CNN	Phase and Intensity	64 × 64	500	230	3.59
**LC-lens (This work)**	FNO	Phase and Intensity	400 × 400	300	15	0.038

Table S1 | Comparison between the convolutional neural network (CNN) and the Fourier neural operator (FNO).

Here we benchmark the performance of various PINN-based phase detection techniques, as summarized in Table S1. Leveraging the strong constraints of the physical model and the superior global capabilities of the Fourier neural network, the proposed system enables simultaneous reconstruction of intensity and phase. Additionally, it achieves the highest reconstruction efficiency, defined as the inference time divided by the image width, with an improvement of nearly two orders of magnitude over CNN-based methods.

Note S5: Comparative Analysis of Reconstruction Efficiency: FNO vs. U-Net

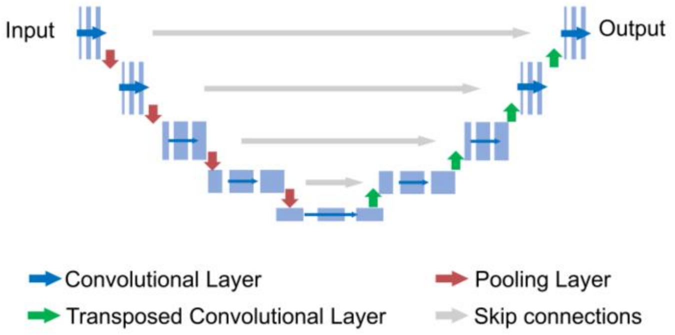

Figure S4 | Architecture of U-Net neural network.

In our framework, the rapid reconstruction of both phase and intensity relies on the combination of the improved FNO architecture and the enhanced physical model. To decouple the contributions of the FNO and the physical model, the U-Net-based algorithm was run on the same hardware configuration as the FNO-based method and was tasked with reconstructing the same dataset.

The architecture of the U-Net neural network is shown in Fig. S4. It features a standard encoder-decoder structure with skip connections. The contracting path consists of five blocks, each with two convolutional layers (with batch normalization and ReLU/Leaky ReLU activations) followed by 2×2 average pooling, increasing the channel depth from 2 to 128. The expansive path utilizes transposed convolutions for upsampling, with feature maps concatenated from the corresponding encoder stage before being processed by two convolutional layers. The final output layer uses a 3×3 convolution to produce two channels, which are scaled by a sinusoidal function and a scaled sigmoid function, respectively.

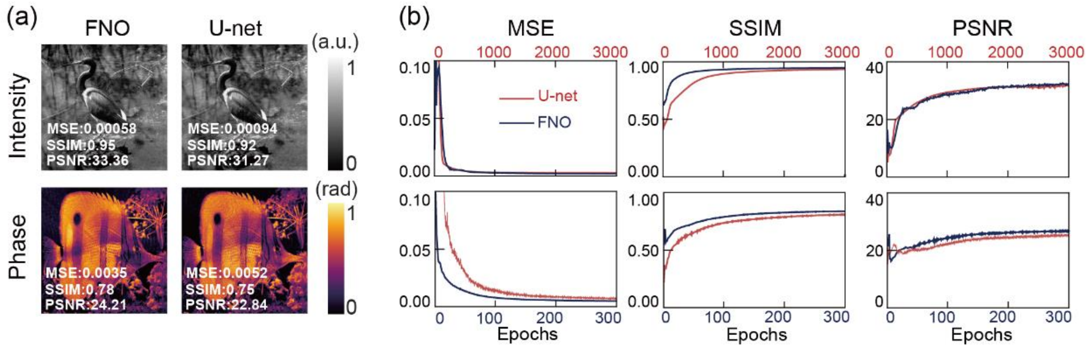

Figure S5 | Reconstruction performance comparison between the FNO-based method and the U-Net-based method. (a) Reconstructed intensity and phase distributions using the FNO-based method and the U-Net-based method, respectively. (b) Evolution of image evaluation metrics for reconstructed intensity and phase with iteration number. MSE: Mean Squared Error; SSIM: Structural Similarity Index Measure; PSNR: Peak Signal-to-Noise Ratio.

As shown in Fig. S5a, the U-Net achieves comparable image quality to the FNO network on the complex amplitude reconstruction task. However, the U-Net-based method required 3000 iterations and approximately 171.7 seconds to converge to a level

equivalent to the FNO network (Fig. S5b), which is an order of magnitude slower in reconstruction efficiency. Therefore, the significant gain in reconstruction efficiency is attributed to the joint utilization of the physical model and the FNO architecture.

Note S6: Bifocal LC-lens design.

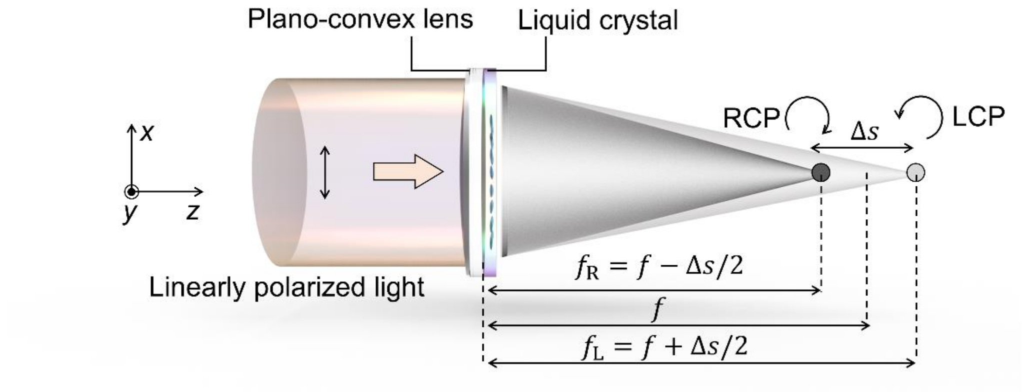

Figure S6 | Schematic of the LC-lens that focuses the circularly polarized light with different focal length.

As shown in Fig. S6, the bifocal LC-lens consists of a liquid crystal layer and a plano-convex lens. The geometric phase induced by the rotation of liquid crystal molecules results in opposite phase distributions for LCP and RCP light. By further incorporating the convergence propagation phase of the refractive lens, the focal points of LCP and RCP light are separated along the optical axis near the original focal point of the plano-convex lens.

The working wavelength of the LC-lens is set to λ = 633 nm. The focal length of the plano-convex lens is f, with the corresponding focal lengths for LCP and RCP light being $f_L = f + \Delta s/2$ and $f_R = f - \Delta s/2$, respectively, separated by a distance Δs along the optical axis. The phase of the plano-convex lens $\phi_{Lens}(x, y)$, which can be expressed as,

$$\phi_{\mathrm{Lens}}(x,y) = \frac{2\pi}{\lambda}(\sqrt{x^2+y^2+f^2} - f). \tag{S12}$$

The required phase distributions for LCP and RCP light, ϕ_{LCP} and ϕ_{RCP}, can be expressed as,

$$\phi_{\mathrm{LCP}}(x,y) = \frac{2\pi}{\lambda}[\sqrt{x^2+y^2+\left(f+\frac{\Delta s}{2}\right)^2} - \left(f+\frac{\Delta s}{2}\right)]. \tag{S13}$$

$$\phi_{\mathrm{RCP}}(x,y) = \frac{2\pi}{\lambda}[\sqrt{x^2+y^2+\left(f-\frac{\Delta s}{2}\right)^2} - \left(f-\frac{\Delta s}{2}\right)]. \tag{S14}$$

The rotation of liquid crystal molecules generates a geometric phase ϕ_{LC}, which exhibits a linear increase from 0 to 2π as the element rotates within the range of 0 to π. Through the geometric phase, a phase $\phi_{LCP} = \phi_{LC}$ is applied to the LCP component of the incident linearly polarized light field, while an opposite phase distribution $\phi_{LCP} = -\phi_{LC}$ is applied to RCP component. Combining Eqs. (S12)-(S14), the geometric phase distribution generated by the liquid crystal layer, ϕ_{LC}, can be calculated as,

$$\phi_{\mathrm{LC}} = |\phi_{\mathrm{Lens}} - \phi_{\mathrm{LCP}}| = |\phi_{\mathrm{Lens}} - \phi_{\mathrm{RCP}}| \approx \frac{\pi}{\lambda}(\frac{f}{\sqrt{x^2+y^2+f^2}} - 1)\Delta s. \tag{S15}$$

The corresponding spatial distribution of the rotation angle θ of the liquid crystal molecules is calculated by,

$$\theta(x,y)=\frac{\phi_{\mathrm{LC}}}{2}=\frac{\pi}{2\lambda}\left(\frac{f}{\sqrt{x^2+y^2+f^2}}-1\right)\Delta s. \tag{S16}$$

The separation distance Δs determines the radial shearing ratio $M_{\mathrm{L}}/M_{\mathrm{R}}$ and defocus diffraction distance $|z_2 - z_1|$ in the physical model. The object and image distances of the complex amplitude imaging system were set to $d_{\mathrm{o}} = d_{\mathrm{i}} = 2f_{\mathrm{L}}$, enabling clear imaging of LCP light with an imaging magnification of $M_{\mathrm{L}} = 1$. The corresponding radial shearing ratio and defocus diffraction distance can be calculated as,

$$\frac{M_{\mathrm{L}}}{M_{\mathrm{R}}}=\frac{d_{\mathrm{i}}-f_{\mathrm{L}}}{d_{\mathrm{i}}f_{\mathrm{L}}}\Big/\frac{d_{\mathrm{i}}-f_{\mathrm{R}}}{d_{\mathrm{i}}f_{\mathrm{R}}}\approx 1+\frac{2\Delta s}{f_{\mathrm{L}}}. \tag{S17}$$

$$|z_2-z_1|=\left|d_{\mathrm{o}}-\frac{d_{\mathrm{i}}f_{\mathrm{R}}}{d_{\mathrm{i}}-f_{\mathrm{R}}}\right|\approx 4\Delta s. \tag{S18}$$

Note S7: LC-lens fabrication.

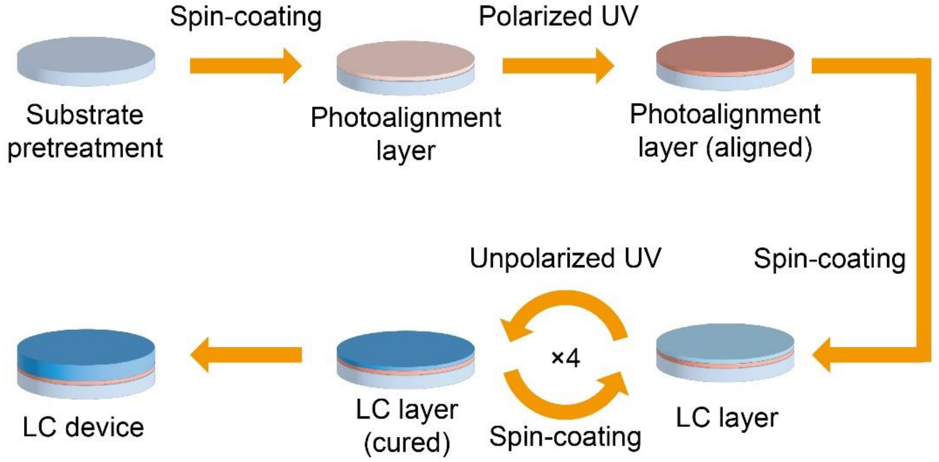

Figure S7 | Schematic of the fabrication process of the LC-lens.

The LC-lens consists of two main components: a plano-convex lens (CX10612-633, Lbtek Optics) and an LC device fabricated on a 1-inch fused silica substrate. The fabrication process of the LC device is shown as Fig. S7 and described in detail below.

First, the fused silica substrate was cleaned with acetone to remove organic contaminants and ensure sufficient cleanliness, followed by a 3-minute UV ozone treatment to improve the surface hydrophilicity. Second, the photoalignment layer (prepared by dissolving 0.15 wt.% SD1 powder in N, N-dimethylformamide, Dainippon Ink and Chemicals Co., Ltd.) was spin-coated onto the pretreated substrate. After coating, the substrate was baked to stabilize the photoalignment layer. Then, the SD1 layer on the substrate was placed on the image plane of the digital micromirror device (DMD)-based microlithography system for exposure. By projecting mask patterns with specific linear polarization states, the SD1 layer records alignment information, thereby guiding the orientation angle distribution of the LC molecules. Specifically, the DMD-generated mask divided the alignment angles uniformly from 0° to 180° in 2° increments. During exposure, each subregion was illuminated with linearly polarized light at a specific angle, achieved by rotating a polarizer accordingly. A 2× objective lens was used during projection to scale the mask pattern, resulting in a final pixel size of 5.4 μm. Due to the limited exposure area, the entire pattern was exposed in two sequential parts (upper and lower sections).

After exposure, the LC solution was spin-coated onto the SD1 alignment layer at 2000 rpm to ensure uniform film formation. The LC solution was prepared by dissolving the LC mixture (Raito Materials Co., Ltd.) in propylene glycol monomethyl ether acetate at a weight concentration of 20%, with oil-bath stirring to accelerate dissolution. Subsequently, the substrate with the LC layer was placed under a unpolarized ultraviolet light source (wavelength 365 nm) for polymerization and curing. The spin-coating and UV curing processes were repeated four times to accumulate the LC layer thickness to meet the half-wave retardation condition, ultimately yielding a high-efficiency LC device.

Note S8: Optimization of the liquid crystal lens using the ray-tracing method.

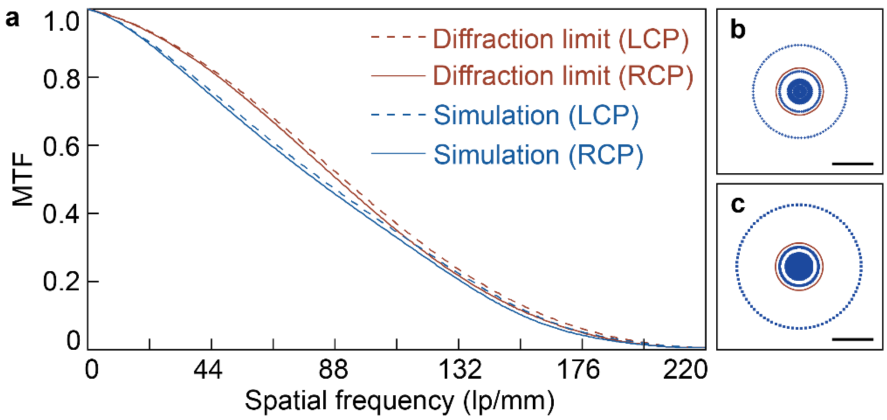

Figure S8 | Simulated modulation transfer function (MTF) curves and spot diagrams under different polarization states. a, MTF curves of the designed LC lens, where red lines represent the diffraction limit, and blue lines indicate the ray tracing simulation results. The dashed and solid lines represent LCP and RCP light, respectively. **b-c,** Simulated spot diagrams of LCP (b) and RCP (c) light. The red circles indicate their corresponding Airy disks. Scale bar: 10 μm.

The planar LC-lens modulates the phase of incident light by leveraging the continuous in-plane variation of the LC molecular orientation. For LCP and RCP light, their phase distributions are opposite in sign. When combined with a plano-convex lens, the LC-lens axially separates the focal points of LCP and RCP light near the original focal point of the plano-convex lens. Here, we use ray tracing method for the design and simulation of LC-lens. The phase profile of Binary 2 can be expressed as:

$$\varphi = M\sum_{i=1}^{N} A_i\rho^{2i}. \tag{S19}$$

where M is the diffraction order set to 1, N is the maximum term specified for the Binary 2 surface, and A_i is the coefficient of ρ^{2i}, where ρ denotes the normalized radial aperture coordinate. By applying the specified optimization and constraint conditions, the optimized results are summarized in Table S2 below. The simulated modulation transfer function (MTF) curves and spot diagrams in Fig. S8 under different polarization states all demonstrate near-diffraction-limited focusing and imaging performance.

**Polarization**	**Normalized Radius (mm)**	**Coeff. on ρ^2**	**Coeff. on ρ^4**	**Coeff. on ρ^6**	**Coeff. on ρ^8**	**Coeff. on ρ^{10}**
**LCP**	1	0.689	-8.288× 10^{-3}	1.063× 10^{-3}	-5.378× 10^{-5}	9.353× 10^{-7}
**RCP**	1	-0.689	8.288× 10^{-3}	-1.063× 10^{-3}	5.378× 10^{-5}	-9.353× 10^{-7}

Table S2 | Optimization results of phase coefficient.

Note S9: Measurement of the PSFs of the LC-lens.

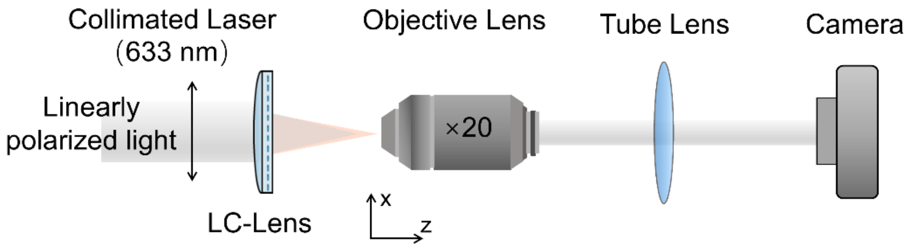

Figure S9 | Schematic of the experimental setup for the measurement of the LC-lens' PSFs.

The experimental setup for characterizing the PSF of the LC-lens is shown in Fig. S9. A linearly polarized laser beam (HSXYD1250, Huashang Laser) at 633 nm was incident on the LC-lens. The focused near field of the LC-lens was subsequently magnified through a 20× microscope system (Mitutoyo, MPLNAPO20XVIR 20×). The PSF was acquired by moving the LC-lens along the optical axis. The measurement results of PSF are shown in Fig. 2a in the main text.

Note S10: Comparison between LC-Lens and complex amplitude imaging metalens.

Name	Aperture size	Cost	Diffraction efficiency	FOV	Spatial resolution (Reconstruction / Theory)
Metalens	2 mm	~30000 RMB	26.47%	50% loss	4.9 μm / 2.9 μm
LC-Lens	10 mm	~9500 RMB	94.23%	No loss	15.88 μm / 15.88 μm

Table S3 | Comparison between LC-Lens and complex amplitude imaging metalens.

Here, we take our previously reported complex amplitude imaging metalens[6] as an example for a comprehensive comparison, as shown in Table S3.

For a 2-mm-diameter Si-based metalens fabricated using expensive electron-beam lithography, the commercial fabrication cost is approximately 30,000 RMB. In contrast, the liquid crystal device with a 10 mm diameter costs about 9,500 RMB. More importantly, current fabrication technology already supports LC devices with diameters up to 100 mm, offering a significant advantage in scalability and cost-effectiveness for large-aperture systems, which is a critical challenge for metasurfaces.

Moreover, due to fabrication errors and optical loss, the diffraction efficiency of the metalens is relatively low (around 26.47%), which is significantly lower than that of the LC-Lens (94.23%).

The metalens relies on spatial multiplexing to capture phase gradients, which directly sacrifices half of the field of view (FOV). The use of non-ideal point spread functions in the metalens also leads to severe degradation in spatial resolution. In contrast, the LC-Lens avoids this trade-off. Crucially, our multiplexing strategy is polarization-based rather than spatial, which is the key factor in preserving the full FOV. Combined with the monocular configuration and the powerful reconstruction capability of the neural network, the LC-Lens system maintains both a wide FOV and high spatial resolution.

Note S11: Calibration of complex amplitude imaging systems.

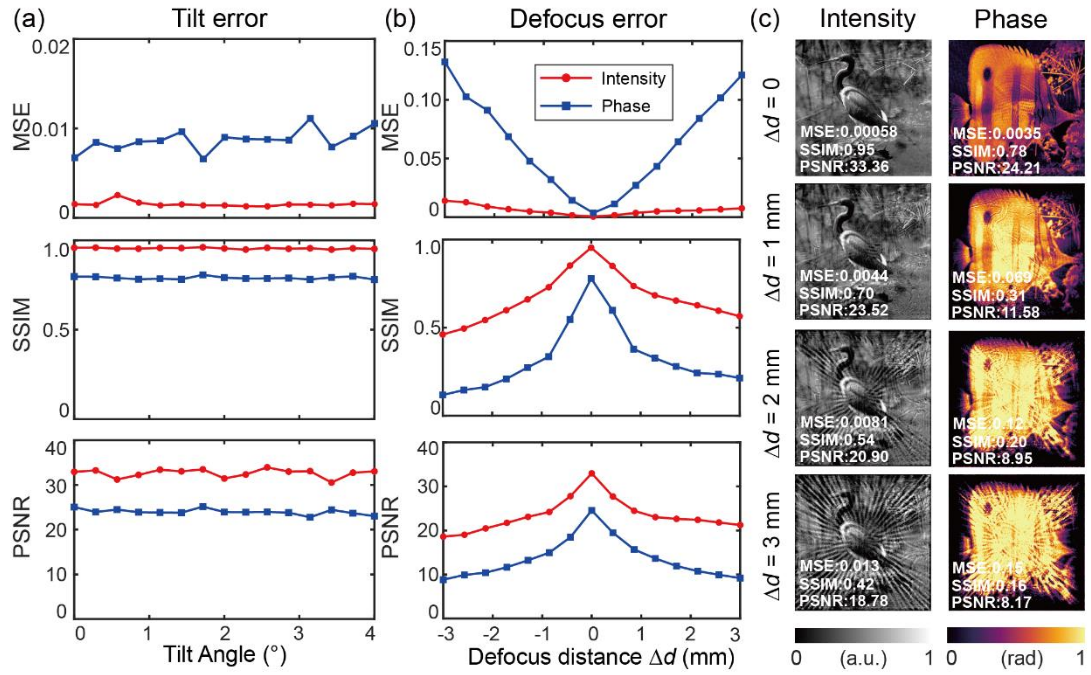

Figure S10 | Simulation of experimental misalignments on the reconstruction performance. (a) Reconstruction error induced by the misalignment tilt of the LC-Lens. (b) Reconstruction error induced by the defocus of the LC-Lens.(c) Complex amplitude reconstruction results under varying defocus distances.

The imperfect alignments in the experimental setup can affect the reconstruction results. Given the simplicity of our optical configuration, which comprises only a single LC-Lens and a polarization sensor, the primary sources of positioning imperfection are the tilt and defocus of the LC-Lens.

We have conducted simulations to evaluate the influence of these misalignments on the reconstruction performance. Identical to the experiment, the object distance in the simulation was set to 15 cm to achieve complex amplitude imaging with 1:1 magnification ratio. As shown in Fig. S10(a), the quality of the reconstructed complex amplitude remains nearly unchanged for LC-Lens tilts within ±4°, as the primary effect is merely a spatial shift of the image. In contrast, the reconstruction algorithm is more sensitive to defocus distance Δd (Fig. S10(b)). Significant image degradation occurs when the image plane deviates by as little as ±3 mm, as shown in Fig. S10(c).

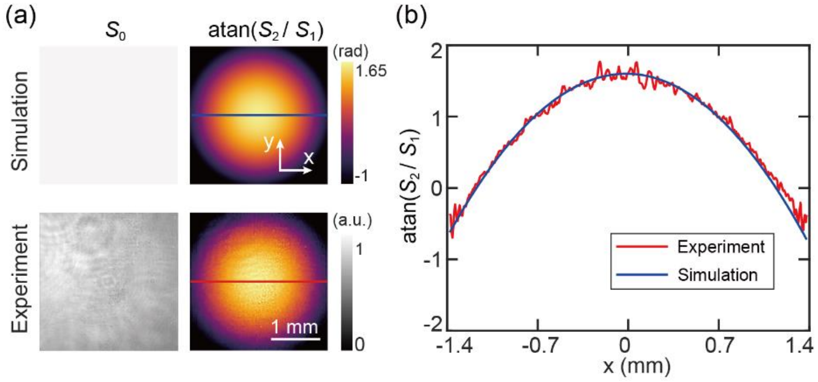

Figure S11 | Comparison between the simulated and measured Stokes parameters. (a) Simulated and measured Stokes parameters under plane-wave illumination. (b) Distribution of the Stokes parameters along the red and blue solid lines marked in (a).

The calibration was achieved by comparing the measured Stokes parameters under plane-wave illumination with the model's predictions. Under coherent, uniform plane-wave illumination, the light wave captured at the image plane carries a quadratic phase term from Fresnel diffraction. At the polarization sensor, the wavefront is transformed into a polarization phase-shift radial shearing interference pattern, resulting in the Stokes parameter distribution depicted in Fig. S11(a). By comparing the experimental and simulated Stokes distributions, the parameters of the optical system can be precisely calibrated. The selected line profiles of the atan(S_2 / S_1), show excellent agreement between simulation and experiment (Fig. S11(b)), which also validates the consistency between the experimental system and the forward physical model.

Note S12: Ideal imaging for the loaded complex amplitude.

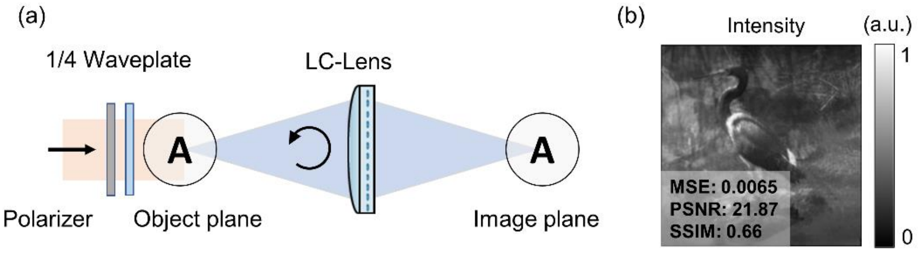

Figure S12 | Ideal imaging for the loaded complex amplitude. a, Schematic of the experimental optical setup for ideal imaging under LCP light illumination. **b**, Captured denoised intensity image.

The use of a 4-*f* system in the complex amplitude generation process with the SLM results in crosstalk of phase image edge information in the reconstructed intensity image. The 4-*f* system is primarily used to isolate the first diffraction order carrying the complex amplitude field, thereby mitigating the effects of the SLM's zero-order diffraction. However, the finite aperture also limits the passage of high-frequency components in the complex amplitude field. In the loaded complex amplitude, abrupt phase transitions correspond to high-frequency information, which is also filtered out, resulting in the presence of phase edge information in the intensity distribution. Additionally, the non-uniform response of the SLM and coherent speckle effects make it challenging to generate high-quality holograms.

To validate the quality of the complex amplitude generated by CGH, the incident light in the complex amplitude imaging setup (Fig. 3(a)) was adjusted to LCP using a linear polarizer and a quarter-wave plate, as shown in Fig. S12(a). In this configuration, the LC-lens demonstrates an ideal single-focal-point PSF for LCP light, allowing for clear imaging of the target object. The denoised intensity image still contains edge information from the phase image (Fig. S12(b)), with MSE, PSNR, and SSIM values of 0.0065, 21.87, and 0.66, respectively. These metrics are comparable to those of the intensity image reconstructed using the liquid-crystal-lens-informed Fourier neural network, demonstrating the high reconstruction fidelity of our computational framework.

Note S13: Measurement of the intensity and phase resolution targets.

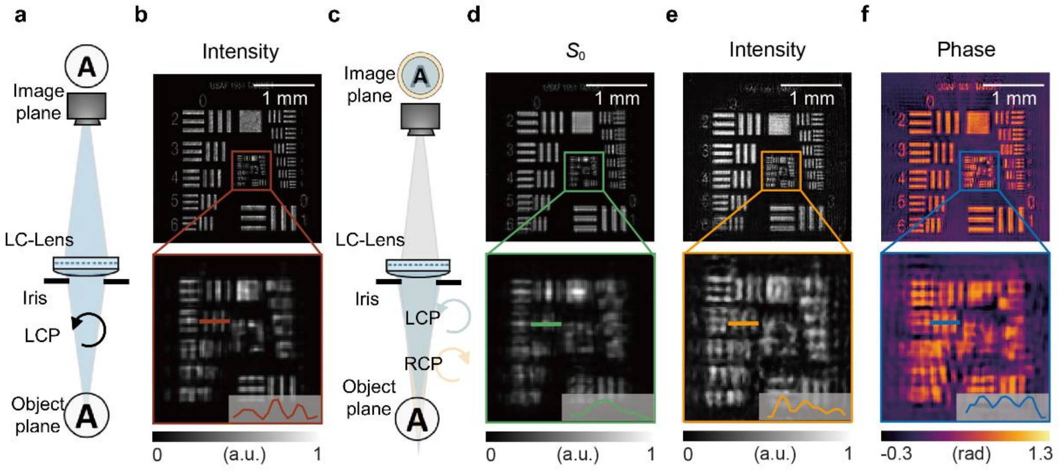

Figure S13 | Measurement of the intensity and phase resolution targets. a, Schematic of the experimental optical setup for ideal imaging under LCP light illumination. **b,** Captured intensity image of the intensity resolution target. **c,** Schematic of the experimental optical setup for ideal imaging under linearly polarized light illumination. **d,** Captured Stokes parameter S_0 of the intensity resolution target. **e-f,** Reconstructed intensity (e) and phase (f) image of the resolution targets.

The spatial resolution of the complex amplitude imaging system includes phase spatial resolution and imaging spatial resolution, which can be measured by a 1951 USAF phase and intensity resolution target, respectively.

To evaluate the spatial resolution of complex amplitude imaging, the 1951 USAF intensity and phase resolution targets were loaded using CGH, respectively. However, due to the limited pixel size of the SLM and the aperture constraints of the 4-*f* filtering system, the minimum linewidth of the generated 1951 USAF resolution target is 15.88 μm, corresponding to Group 2, Element 3. To match the resolution target, the aperture of the LC-lens was restricted to 3.7 mm using an iris. At a 1:1 imaging magnification, the theoretical minimum resolvable linewidth is 15.85 μm, consistent with the minimum linewidth of the resolution target.

Experimentally, the 1951 USAF intensity resolution target was loaded using CGH. The clear imaging of the intensity resolution target with LCP illumination (Fig. S13(a)) is shown in the Fig. S13(b). For complex amplitude imaging, as shown in Fig. S13(c), the incident light is linearly polarized, and the LC-lens simultaneously captures the LCP and RCP components at two distinct image distances. With such a non-ideal bifocal PSF, the intensity image (Fig. S13(d)) captured by the imaging system, corresponding to the Stokes parameter S_0, is blurred and fails to resolve Group 2, Element 3. In contrast, the intensity image reconstructed by the neural network resolves Group 2, Element 3, with no loss of spatial resolution. Subsequently, a 1951 USAF phase target with a height of 1.2 rad was loaded to evaluate the spatial resolution of the phase reconstruction, with results indicating no degradation in resolution. Thus, despite the non-ideal bifocal PSF of the LC-lens, the neural network effectively restores the imaging performance to its ideal state.

Note S14: Dynamic complex amplitude imaging for gas flow and flame combustion.

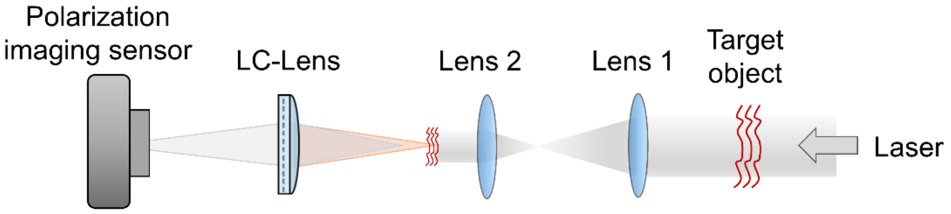

Figure S14 | Schematic of the experimental setup for the dynamic complex amplitude imaging.

As shown in Fig. S14, the experimental setup consists of the fabricated LC-lens cascaded with a 4-*f* system to enable dynamic complex amplitude imaging with an expanded FOV. Lenses 1 and 2 have focal lengths of 100 mm and 30 mm, respectively, corresponding to an imaging magnification ratio of 0.3. Subsequently, the LC-lens performs cascaded imaging at a 1:1 magnification ratio, resulting in an expanded FOV of 9.2 × 9.2 mm^2 for the entire imaging system.

References

1. Shannon CE. Communication in the Presence of Noise. *Proceedings of the IRE* 1949; **37**: 10-21.
2. Wang F, Bian Y, Wang H *et al.* Phase imaging with an untrained neural network. *Light Sci Appl* 2020; **9**: 77.
3. Tu H, Liu H, Pan T *et al.* Deep empirical neural network for optical phase retrieval over a scattering medium. *Nat Comm* 2025; **16**: 1369.
4. Bai C, Peng T, Min J *et al.* Dual-wavelength in-line digital holography with untrained deep neural networks. *Photonics Research* 2021; **9**: 2501-2510.
5. Chen Q, Huang D, Chen R. Fourier ptychographic microscopy with untrained deep neural network priors. *Opt Express* 2022; **30**: 39597-39612.
6. Li L, Wang S, Zhao F *et al.* Single-shot deterministic complex amplitude imaging with a single-layer metalens. *Sci Adv* 2024; **10**: eadl0501.
